# *INSIG2* is Associated with Lower Gain in Weight-for-Length Between Birth and Age 6 Months

**DOI:** 10.4137/cmped.s2279

**Published:** 2009-02-18

**Authors:** Ann Chen Wu, Matthew W. Gillman, Elsie M. Taveras, Augusto A. Litonjua

**Affiliations:** 1 Center for Child Health Care Studies, Department of Ambulatory Care and Prevention, Harvard Pilgrim Health Care and Harvard Medical School, Boston, Massachusetts, U.S.A.; 2 Division of General Pediatrics, Children’s Hospital, Boston, Massachusetts, U.S.A.; 3 Department of Nutrition, Harvard School of Public Health, Massachusetts, U.S.A.; 4 Channing Laboratory, Department of Medicine, Brigham and Women’s Hospital.; 5 Harvard Medical School, Boston, Massachusetts, U.S.A.; 6 Center for Genomic Medicine, Department of Medicine, Brigham and Women’s Hospital, Boston, Massachusetts, U.S.A.

**Keywords:** *INSIG2*, obesity, infancy, weight-for-length

## Abstract

Researchers have described the association of a common DNA polymorphism, rs7566605, near *INSIG2* (insulin-induced gene 2) with obesity in multiple independent populations that include subjects ages 11–60 years.^1^ To our knowledge, no studies have examined the association of this polymorphism with weight status during early childhood. We explored the association of the rs7566605 polymorphism with weight-for-length among 319 children at 6 months and 3 years participating in Project Viva, a pre-birth cohort study. In contrast to studies of older individuals, CC homozygosity was associated with lower gain in weight-for-length z-score between birth and age 6 months than GG homozygosity or GC heterozygosity. At age 3, we did not find an association. The association of *INSIG2* gene with obesity may change direction with age.

## Background

Obesity is associated with multiple severe chronic illnesses such as cardiovascular disease, type 2 diabetes mellitus, metabolic syndrome, hypertension, stroke, and some forms of cancer.

Recent studies have suggested that the rate of weight gain during infancy is important in the development of obesity.^2^ Rapid weight gain during the first 4 to 6 months of life, perhaps even during the first week of life,^3^ appears to be associated with higher risk of obesity in childhood and adulthood.^2^,^4^,^5^

The single nucleotide polymorphism, rs7566605 is 10 kb upstream of the *INSIG2* gene. In a study of subjects ages 11 to 60 years, Herbert et al. found that the mean BMI of rs7566605 CC homozygotes under a recessive model was 0.60 kg/m^2^ higher than for GC or GG genotypes (p = 0.0008).^1^ Since then, some studies have replicated these results^6^–^11^ while other studies have not.^12^–^20^ Despite the finding in a few studies that *INSIG2* is not associated with the risk for obesity, the finding of a positive association in five cohorts is unlikely a false positive finding.^18^–^21^ No study to date, however, has addressed this issue in young children.

The objective of this exploratory study was to determine the extent to which an association exists between the polymorphism rs7566605 and gain in adiposity in the first 6 months and the first 3 years of life. We hypothesized that CC homozygosity would be associated with greater gain.

## Methods

Project Viva is a cohort study of pre- and perinatal factors related to outcomes of pregnancy and child health. Details of recruitment have been previously published.^22^ Of 2128 children born to mothers participating in Project Viva, 1579 children were eligible for the age 3 year in-person follow up visit, and we were successful in visiting 1294 of them. Of these, 835 children were Caucasian (385 were non-Caucasian, and 74 were missing race/ethnicity data), 671 had blood samples, and 521 of their parents provided consent for genetic testing. Of 521 children, 319 have results for the rs7566605 polymorphism. This study was approved by the human subjects committees of Harvard Pilgrim Health Care, Brigham and Women’s Hospital, and Beth Israel Deaconess Medical Center.

We compared the 319 Caucasian children in our study to the Caucasian children who were not included. We noted no material differences between the two groups for child’s gender, birthweight, gestational age, weight-for-length at birth, weight-for-height at age 3 years, maternal pre-pregnancy BMI, paternal BMI, maternal smoking during pregnancy, maternal age, or maternal history of gestational diabetes. The mean weight-for-length z-score at age 6 months of 0.62 [SD 0.99] for subjects in our study was smaller than for subjects who were not in our study (0.79 [SD 0.88], p = 0.02).

We obtained birth weight from the medical record. At ages 6 months and 3 years, study research assistants weighed the children with a digital scale (Seca Model 881, Seca Corporation, Hamburg, Germany), and measured length a few days after birth and at 6 months and height at 3 years using a Shorr measuring board (Shorr Productions, Olney, MD).

The research assistants collected additional demographic information on social, economic, maternal and paternal health information through self-administered questionnaires and interviews at study visits during pregnancy, post-delivery, and at the 6-month and age 3-year visits.

Our main outcome measure was weight-for-length z-score, a marker of adiposity in this age group.^23^,^24^ We used 2000 CDC growth charts to calculate weight-for-length z-scores for each time point.^25^,^26^ We adjusted weight-for-length z-scores at 6 months (and 3 years) for weight-for-length z-score at birth, which is algebraically equivalent to examining change from birth to 6 months (or 3 years) in weight-for-length z-score. Obesity is commonly measured by body-mass-index (BMI) in older children and adults, but weight-for-length is a better measure in children under the age of 2 years and is applicable to age 3.^23^

We genotyped the *INSIG2* genetic variant rs7566605 with the TaqMan assay (Applied Biosystem, CA), and the genotyping completion rate was >95%, with less than 1% discordance.

## Statistical Analysis

We conducted analyses in SAS version 9.1 (SAS Institute, Cary, NC, 2002–2003). In bivariate analyses, we evaluated each variable according to genotype. To estimate the effect of being CC homozygous for *INSIG2* on weight–for-length at birth, 6 months, and 3 years, we used multivariate linear regression assuming a recessive model. Our final multivariate model included gender and candidate variables which confounded the main effect by >10%.

## Results

Genotype frequencies (13% CC, 46% GC, 40% GG) were similar to the frequencies in other populations and consistent with Hardy-Weinberg equilibrium (p = 0.99).

The variant allele (C) frequency was 0.37. Subjects who were CC homozygous for rs7566605 did not differ from subjects who were GC or GG carriers for gestational age, gender, maternal education, or family income (Table 1). Children who were CC homozygous for rs7566605 experienced shorter breastfeeding duration (4.5 months versus 7.3 months) and had mothers with slightly lower pre-pregnancy body mass index (23.3 versus 24.0 kg/m^2^ for GC or GG carriers). Subjects who were CC homozygous were more likely to have been born to a mother with a hypertensive disorder during pregnancy, including chronic hypertension, pre-eclamsia, or gestational hypertension (p = 0.02).

As shown in Table 1, subjects who were homozygous for the C allele had lower mean weights than the other subjects at ages birth and 6 months, although this finding did not reach statistical significance. For CC homozygotes, mean birthweight was 3448 g [SD 487] compared with 3589 g [SD 527] in subjects possessing GC or GG. Compared with GC or GG carriers, the mean weight-for-length z-scores for subjects who were CC homozygous were similar at birth, appeared to be somewhat lower at 6 months, but slightly higher at age 3 years.

On multivariable linear regression, at age 6 months, subjects who were CC homozygous had weight-for-length z-scores that were 0.71 less (95% CI −1.18, −0.25, p = 0.0027) than GC and GG carriers when adjusting for gender, maternal pre-pregnancy BMI, weight-for-length at birth, maternal history of hypertensive disorder during pregnancy, and duration of breastfeeding in months (Table 2). By age 3 years, the weight-for-length was similar regardless of genotype.

## Discussion

Our exploratory study suggests *INSIG2* CC homozygosity is associated with smaller gain in weight-for-length between birth and age 6 months, but that by the age of 3 years no association is present. The finding of lower gain in adiposity during infancy associated with CC was contrary to our hypothesis. One possibility is chance. Our sample size was relatively small for a genetic association study, and our results will need to be validated in other cohorts. A second possibility is that low weight gain in infancy is a precursor to later obesity, and CC homozygosity underlies both. However, this possibility is unlikely because although one study suggests low infancy weight gain is associated with later coronary artery disease,^27^ no study shows that relation with later obesity per se. In fact, most studies suggest the opposite: rapid weight gain in infancy is directly associated with later obesity.^3^–^5^ A third possibility is that CC homozygosity is associated with lower adiposity in very early life but greater adiposity later in childhood or adulthood. Our finding of null results at age 3 years is consistent with this interpretation. Without knowing more about the function of the INSIG2 gene, it is difficult to speculate further about this possibility. Future studies with data from infancy through age 11 years, the youngest age of previous studies, would be useful.

This is the first study to examine the *INSIG2* gene in the first three years of life. Strengths of this study include accurate measures of length and weight, which is critical in studies of infancy.^28^ Similar to other genetic association studies, our study has limitations of potential population stratification because different ethnic/racial groups possess different allele frequencies. However, we limited our analysis to Caucasian children, limiting the possibility of this potential problem. Our analyses also found that the mothers of children who were CC homozygous were more likely to have a hypertensive disorder during pregnancy and less likely to be breastfed; this association has not been studied in the literature and may be a true association or due to chance. On the other hand, *INSIG2* may be associated with maternal hypertensive disorders, and the presence of these disorders makes the mothers less likely to breastfeed. Since we did not genotype the mothers, these associations will need to be studied in other cohorts.

In conclusion, gain in adiposity in the first six months of life may be influenced the *INSIG2* intronic polymorphism rs7566605. The effect of this gene on weight may change direction with age.

## Figures and Tables

**Table 1:** Characteristics of 319 children participating in Project Viva.

	**RS7566605 CC (n = 43)**	**RS7566605 GC or GG (n = 276)**	**p-value**
**Parental Characteristics**			
Mean age [SD] (years)	32.1 [4.4]	33.4 [4.5]	0.08
Maternal Mean pre-pregnancy BMI [SD] (kg/m^2^)	23.3 [3.6]	24.0 [4.7]	0.30
Paternal Mean pre-pregnancy BMI [SD] (kg/m^2^)	27.1 [3.4]	26.4 [4.1]	0.34
Gestational weight gain^*^ (n = 315)			0.88
Excessive	21 (50%)	139 (51%)	
Adequate	16 (38%)	95 (35%)	
Inadequate	5 (12%)	39 (14%)	
Maternal history of hypertensive disorders during pregnancy^†^			0.02
None	34 (81%)	249 (91%)	
Chronic hypertension	1 (2%)	0 (0%)	
Pre-eclamsia	2 (5%)	10 (4%)	
Gestational hypertension	5 (26%)	14 (5%)	
Maternal history of gestational diabetes^‡^			0.09
None	31 (74%)	233 (85%)	
Gestational diabetes	2 (5%)	14 (5%)	
Impaired glucose tolerance	9 (21%)	27 (10%)	
Feeding status at 6 months			0.002
Formula only	10 (23%)	20 (7%)	
Weaned	17 (40%)	93 (34%)	
Mixed	11 (26%)	79 (29%)	
Breastmilk only	5 (12%)	81 (30%)	
Mean breastfeeding duration [SD] (months)	4.5 [4.3]	7.3 [ 4.5]	0.0001
Smoking during pregnancy (n = 313)	4 (10%)	24 (9%)	0.89
Marital Status			0.46
Married	42 (98%)	258 (93%)	
Cohabitate	0 (0%)	6 (2%)	
Divorced or Separated	1 (2%)	6 (2%)	
Other	0 (0%)	6 (2%)	
Household Income at enrollment			0.85
$10,000–$20,000	0 (0%)	2 (0.7%)	
>$20,000–$40,000	3 (7%)	15 (6%)	
>$40,000–$70,000	10 (24%)	53 (20%)	
>$70,000	29 (69%)	198 (73%)	
Don’t know	0 (0%)	3 (1%)	
Mother’s education			0.57
Less than high school or high school graduate	3 (7%)	9 (3%)	
Some college or college degree	25 (58%)	166 (60%)	
Graduate degree	15 (35%)	101 (37%)	
**Child Characteristics**			
Mean weight [SD] (grams)			
Birth	3448 [487]	3589 [527]	0.10
Birthweight category			0.16
<2500 grams	0 (0%)	0 (0%)	
2500–4000 grams	38 (88%)	219 (79%)	
>4000 grams	5 (12%)	57 (21%)	
Mean weight for length z-score [SD]			
Birth	0.40 [0.64]	0.49 [0.73]	0.55
6 months	0.29 [0.87]	0.66 [0.99]	0.05
3 years	0.45 [0.94]	0.37 [1.0]	0.67
Mean gestational age [SD] (weeks)	39.7 [1.6]	39.7 [1.7]	0.94
Female gender	20 (47%)	140 (51%)	0.61

* Categories of gestational weight gain according to guidelines of the Institute of Medicine.^29^

† Hypertensive disorders of pregnancy were categorized using clinical blood pressure and urine protein measurement. Women were categorized as having preeclampsia if they did not have chronic hypertension but developed increased blood pressure and proteinuria (dipstick value of 1+ on two or more occasions or >2+ once) >4 hours but <7 days apart, or if she had chronic hypertension and developed proteinuria after 20 weeks of gestation.^30^

‡ Women were routinely screened for gestational diabetes at 26–28 weeks gestation with a non-fasting oral glucose challenge test in which venous blood was sampled 1 hour after a 50-g oral glucose load. If the 1-hour glucose result was at least 140 mg/dL, the participant was referred for a 100-g fasting glucose 3-hour tolerance test. Normal results were blood glucose below 95 mg/dL at baseline, below 180 mg/dL at 1 hour, below 155 mg/dL at 2 hours, and below 140 mg/dL at 3 hours.^31^ We categorized participants with a normal screening glucose challenge as having normal glucose tolerance; those who failed the challenge test but had 0 or 1 abnormal result on the fasting glucose tolerance test as having impaired glucose tolerance, and those who had at least two abnormal results as having gestational diabetes.^29^

**Table 2:** Associations of CC homozygosity at rs7566605, compared with GC or GG, with weight-for-length. Results based on linear regression while adjusting for gender and maternal pre-pregnancy body mass index, weight-for-length at birth (except for birth), duration of breast-feeding (in months), and of a maternal hypertensive condition during pregnancy (pre-eclamsia, chronic hypertension, or pregnancy induced hypertension).

	**Estimate**	**95% Confidence intervals**	**p-value**
Birth	−0.20	−0.54–0.13	0.24
6 months	−0.72	1.18–0.25	0.0027
3 years	−0.25	−0.71–0.20	0.27
